# Predicting Factors for Requiring Routine Postoperative Blood Analysis in Primary Hip and Knee Arthroplasty: A Prospective Study

**DOI:** 10.7759/cureus.39283

**Published:** 2023-05-21

**Authors:** Herbert Gbejuade, Sharan Sambhwani, Robert M Ubsdell, Maurice Dungey, Ashan Kandiah, Srinivasan Shyamsundar

**Affiliations:** 1 Orthopaedics, Kettering General Hospital, Kettering, GBR; 2 Orthopaedics, University Hospitals Leicester, Leicester, GBR

**Keywords:** bun, complete blood count, blood test, post-operative management, urea and electrolytes, full blood count, primary total hip arthroplasty, knee replacement arthroplasty

## Abstract

Background

Minimising unnecessary expenditure is essential to cope with high demands on the health sector. A set of full blood count, electrolyte, creatinine and urea tests cost £12 in the National Health Service (NHS). Identifying selected patients requiring postoperative blood tests following primary knee and hip arthroplasty will avoid unnecessary tests and help to reduce expenditure.

The aim of our study is to propose criteria for requesting postoperative blood tests that are safe and do not miss patients.

Materials and methods

We prospectively evaluated 126 patients (72 in the total knee replacement (TKR) group and 54 in the total hip replacement (THR) group) who underwent either an elective primary THR or a TKR. The mean patient age was 71 years. Patient demographics as well as in-patient events throughout each patient’s hospital stay were recorded. Hospital readmissions were also monitored for up to 90 days postoperatively.

Statistical analysis was performed using SPSS Statistics software (IBM Corp., Armonk, NY) with paired t-tests / Wilcox and mixed measures analysis of variance. Binary logistic regression was used to identify predictors of patients requiring a postoperative blood test.

Results

Analysis of our data identified the following as risk factors for requiring postoperative full blood count tests, including pre-operative Hb of ≤ 110 g/L, cardiac disease, clinical features of anaemia postoperatively and intraoperative blood loss of > 500 mL. The additional risk factors identified for requiring postoperative electrolyte and urea tests are deranged pre-operative electrolytes and clinical signs or symptoms of electrolyte/renal disturbance such as anuria. No patient was readmitted within 90 days of discharge.

Conclusion

Overall, applying the criteria we have devised would have saved 74 blood tests in the cohort of 126 patients. This provides an odds ratio of 14.0 (95% confidence interval: 1.77-110, p=0.012) of an abnormal result in the patients that would have been tested, compared to those that would not have been tested.

## Introduction

Introduction

Hip and knee arthroplasties are two of the most frequently performed procedures in elective orthopaedics [1] with over 160,000 total hip replacement and total knee replacement procedures performed each year in England and Wales [2]. Now that people are living longer, the number of these procedures performed is predicted to increase [3]. With this in mind, the health care expenditure would also be expected to increase. Therefore, improving efficiency in healthcare to accommodate demand whilst maintaining a high standard of care must be a top priority. The implementation of enhanced recovery should produce a reduction in the length of hospital stay without compromising health care [4,5]. A measure that can aid enhanced recovery and cost efficiency in the health service is to minimise unnecessary investigations in hospitals [6].

Preoperative assessment is a mandatory prerequisite in many health institutions for patients scheduled for elective surgery. This includes blood analyses which enable prompt optimisation of patients ahead of their planned elective procedures.

Patient optimisation continues in the operating theatre with intraoperative measures such as advancement in anaesthetic monitoring, minimising blood loss with the use of anti-thrombolytics [7-9], cell salvage and bedside blood testing kits in order to ensure that the patient is adequately monitored and managed throughout their surgery [10-13].

Despite all the aforementioned strategies, it is still common to perform postoperative blood analyses as a matter of routine, which is commonly in the form of full blood count (FBC), and urea and electrolytes (U&Es). These are commonly performed on the first or second postoperative day. This can delay patient discharge from the hospital, may cause undue patient anxiety and incur unnecessary costs, especially when there are no clear clinical indications to request these tests. In addition, this may hamper the current strive towards enhanced recovery and same-day discharge of patients following lower limb arthroplasty [14]. Moreover, a set of full blood count, electrolyte, creatinine and urea tests cost £12 in the NHS [6,15].

In view of our aims, we therefore prospectively evaluated patients who underwent elective total hip replacements (THRs) and total knee replacements (TKRs). Our primary outcome is the identification of risk factors for abnormal FBC and U&E; while the secondary outcome is hospital revisit or/and readmission for electrolyte derangement within 90 days of their discharge following their index arthroplasty procedures.

## Materials and methods

This is a prospective study that evaluated patients who underwent either a primary total knee replacement (TKR) or a total hip replacement (THR) between October 2019 and January 2020. The project was registered with our clinical governance department as a service evaluation project. Patients who had arthroplasty following a fracture, revision arthroplasty procedures and uni-compartmental knee replacements were excluded from this analysis.

Parameters recorded include patient age, gender, planned procedure, relevant comorbidities, regular medication, preoperative and postoperative haemoglobin, platelets, sodium, potassium, urea, creatinine, and estimated glomerular filtration rate (EGFR). Intraoperative blood loss, tourniquet time (in TKR cases) and intraoperative fluid administration were recorded. Episodes of acute kidney injury (AKI) were defined as a change in creatinine of >150 % or an increase of >26.5 mmol/L [16].

Patients that required intervention for deranged postoperative blood results were noted. In our unit, the threshold for blood transfusion is haemoglobin level (Hb) <70 g/L; or <80 g/L with cardiac disease or symptoms such as dizziness, hypotension or active bleeding. The threshold for intervention in platelets was defined as <100 (x109/L). Electrolyte abnormalities were defined as outside of the normal laboratory reference ranges.

When grouping comorbidities, coronary heart disease and valvular heart disease were combined as ‘cardiac disease’; atrial fibrillation was listed separately. All patients were administered intraoperative tranexamic acid as per standard practice in our unit. In addition, all patients were commenced on enoxaparin six hours postoperatively.

Statistics

Statistical analysis was performed using Statistical Package for Social Sciences (SPSS v. 25, IBM Corp, Armonk, NY). Data were assessed for normality and were presented as mean ± standard deviation or median (interquartile range) as appropriate. Pre-to-post-operative changes were assessed via paired t-tests or Wilcoxon signed ranks for parametric and non-parametric data respectively. Differences between hybrid and fully-cemented total hip replacements were examined via independent t-tests or Mann-Whitney U tests as appropriate. Mixed measures analysis of variance (ANOVA) was used to assess pre-to-post changes between males and females and between the >70 years and <70 years old age groups. Binary logistic regression was performed looking for predictors of patients requiring a post-operative blood transfusion or with post-operative AKI or abnormal electrolytes. Odds ratios with 95% confidence intervals were used to assess the effectiveness of the proposed algorithms. Statistical significance was defined as P <0.05.

## Results

A total 126 consecutive total joint replacements were included in this study (72 in TKR group and 54 in THR group). Of the THR group, 46.3% were hybrids while 53.7% were fully cemented. No significant statistical differences were found between these two types of total hip replacements, hence were grouped together as 'total hip replacements’ (THR). 

Overall, there were twice as many females (83) than males (43) and the mean patient age was 71 years (range 51-96 years). The patient demographics including major co-morbidities and anticoagulant medication are outlined in Table 1. The mean intra-operative blood loss was higher with the hybrid total hip replacement compared to the fully cemented total hip replacement (hybrid: 390ml vs fully cemented: 325ml) but this was not statistically significant. 

**Table 1:** Patient demographics Data are presented as mean (range) or number (percentage) as appropriate. AF=atrial fibrillation; COPD=chronic obstructive pulmonary disease; VTE=venous thromboembolic; N/A=not applicable; ASA=American Society of Anaesthesiologists

Parameter	TKR (n=72)	THR (n=54)
Sex		
Male	26 (36.1%)	17 (31.5%)
Female	46 (63.9%)	37 (68.5%)
Age (y)	71.7 (52 - 96.5)	70.2 (51-87.4)
ASA		
I	3 (4.2%)	4 (7.4%)
II	39 (54.2%)	36 (66.7%)
III	30 (41.7%)	4 (25.9%)
IV	0 (0%)	0 (0%)
Co-morbidities		
Cardiac disease	24 (33.3%)	7 (13.0%)
AF	3 (4.2%)	3 (5.6%)
COPD	2 (2.8%)	3 (5.6%)
Diabetes mellitus	7 (9.7%)	2 (3.7%)
Previous VTE event	4 (55.6%)	2 (3.7%)
Preoperative anticoagulant / antiplatelet		
Aspirin	11 (15.3%)	4 (7.4%)
Clopidogrel	3 (4.2%)	0
Warfarin	0	2 (3.7%)
Apixaban	5 (6.9%)	2 (3.7%)
Rivaroxaban	0	2 (3.7%)
Tourniquet time (min)	81 (49-116)	N/A
Intraoperative blood loss (mL)	35 (5-250)	330 (90-1000)

Haemoglobin (Hb) 

As expected, Hb significantly decreased post-operatively after both THR and TKRs (Table 2). In the TKR group, no patient had a postoperative drop in Hb significant enough to reach the threshold of transfusion. However, two patients (2.8%) had a postoperative Hb <90 g/L (Case 1: pre-op Hb 109 g/L to post-op 87 g/L; Case 2: Hb 120 g/L to 87 g/L); both were asymptomatic. 

**Table 2:** Blood parameters Data presented as mean ± standard deviation. * Denotes non-normally distributed data [Median (Interquartile range)] † Denotes significant difference between pre-op and post-operative value using paired t-tests or Wilcoxon for parametric and non-parametric data respectively (P < 0.05). eGFR=estimated glomerular filtration rate

Blood parameter	Pre-op	Post-op	Normal range
TKR (n=72)			
Haemoglobin (g/L)	133 ± 14.8	115 ± 13.3 †	130-180
Platelets (x109/L)	279 ± 71.1	245 ± 60.4 †	150-450
Sodium* (mmol/L)	140 (139-142)	137 (135-138) †	133-146
Potassium (mmol/L)	4.47 ± 0.46	4.17 ± 0.44 †	3.5-5.3
Urea* (mmol/L)	5.85 (4.80-6.90)	5.60 (4.68-8.1)	2.9-8.1
Creatinine (mmol/L)	77.2 ± 17.4	80.4 ± 21.2 †	60-120
eGFR* (mL/min/1.73m2)	76.3 (66.2-90)	75.5 (63.4-90)	> 90
THR (n=54)			
Haemoglobin (g/L)	137 ± 13.4	113 ± 16.3 †	130-180
Platelets* (x109/L)	253 (228-306)	237 (193-277) †	150-450
Sodium* (mmol/L)	141 (139-141)	137 (135-139) †	133-146
Potassium (mmol/L)	4.32 ± 0.36	4.11 ± 0.44 †	3.5-5.3
Urea* (mmol/L)	5.05 (4.08-5.78)	5.40 (4.53-6.58)	2.9-8.1
Creatinine* (mmol/L)	67.0 (60.3-81)	68.5 (62.0-84.8)	60-120
eGFR* (mL/min/1.73m2)	84.8 (76.8-90.0)	83.2 (74.3-90.0)	> 90

In the THR group, one patient had a postoperative drop in haemoglobin which reached the threshold of transfusion (change of Hb from 113 g/L to 74 g/L postoperatively with cardiac comorbidity). The patient was noted to have had an intraoperative blood loss of 350 ml. Four other patients had postoperative Hb <90 g/L, but none of these reached the threshold of transfusion. Therefore, in total, five THR patients (9.3%) had a postoperative Hb <90 g/L. Binary logistic regression found no significant predictors of requiring blood transfusion or having a post-operative Hb <90 g/L (P >0.05). 

The change in Hb in the THR group was associated with intraoperative blood loss (r=-0.596, P <0.001); i.e., the greater the blood loss, the greater the reduction in Hb. There was no similar significant association in the TKR group (r=-0.141, P=0.238). 

In the THR group, mixed measures ANOVA revealed a significant change in Hb from pre-op to post-op (F=205, P < 0.001). There was also an interaction between gender and change in Hb (F=4.858, P=0.032). The change in haemoglobin was significantly greater in females (-26.2 ± 11.2 g/L) than in males (-19.2 ± 10.1 g/L), suggesting that female patients are likely to have a greater susceptibility to low post-operative Hb. 

In the TKR group, there was a significant change in Hb from pre-op to post-op (F=245, P <0.001) in both males (140 ± 14.7 to 121 ± 14.9 g/l) and females (129 ± 13.7 to 112 ± 11.2 g/L); but no interaction effect was found (F=0.377, P=0.541). 

In both THR and TKR groups, no effect of age on Hb was observed in the TKR group (P=0.661) or the THR group (P=0.806) 

Platelets 

No patient in the TKR or THR group had a significant drop in platelets to the threshold required for intervention. 

Renal function and electrolytes 

In the TKR group, six of 72 (8.3%) patients had abnormal postoperative electrolytes (Table 2). Two of these six had pre-existing abnormal electrolytes and a further two had pre-existing low-normal electrolyte levels. In addition, two of the 54 patients in the TKR group had postoperative acute kidney injuries (AKI). 

In the THR group, three of the 54 (5.6%) patients had abnormal postoperative electrolyte results. Of these three, two patients had postoperative hypokalaemia and the third had postoperative hyponatraemia. In addition, three of the 72 patients in the TKR group had postoperative AKI. 

Binary logistic regression for predicting the presence of an electrolyte abnormality after TKR found that pre-operative sodium was a significant predictor (B=-0.242, P=0.045). Pre-operative haemoglobin was the next closest factor to significance (P=0.053). 

For THRs, no factors were significant in predicting a post-op electrolyte abnormality, with pre-operative potassium being the closest factor to significance (p=0.053). Binary logistic regression for predicting AKI found pre-operative haemoglobin as a significant predictor in THRs (P=0.030) and close to significance in TKRs (p=0.069), but no other factors were close to significance. 

In both TKR and THR groups, males had higher baseline creatinine levels than females (P=0.001 and P=0.014 respectively). No interaction was between sex and change in creatinine in TKR (F=0.105, P=0.747) or THR (F=0.093, P=0.761) 

There were no effects of gender on change in sodium in either procedure (TKR P=0.907; THR P=0.607) or change in potassium (TKR P=0.792; THR P=0.535). Likewise, in both groups, there were no effects of age on creatinine (TKR: P=0.471; THR: P=0.545), change in sodium (TKR P=0.236, THR P=0.231), or change in potassium (TKR P=0.850, THR P=0.625).

American Society of Anesthesiologists (ASA) grading 

No significant difference was found in the ASA grades between TKR and THR (χ2=3.583, P=0.167). Also, there was no difference in the ASA grades between fully cemented and hybrid THR (χ2=1.97, P=0.374). 

ASA was found not to be a significant predictor of treating U&Es, abnormal Hb, or AKI. 

90-day hospital readmission rates

We monitored our cohort of patients for 90 days following their hospital discharge after their index procedures. We found no cases of hospital readmission due to derangements in full blood count, urea or electrolyte that could be related to their previous admissions for their arthroplasty procedures. 

Predictors of abnormal postoperative results 

Applying Table 3 criteria to our THR cohort would have led to performing 18 full blood count tests postoperatively (instead of 54) saving 36 postoperative blood tests. Similarly, applying the same criteria to the TKR group would have led to 34 postoperative full blood count tests (instead of 72), saving 38 tests. 

**Table 3:** Proposed criteria for requesting postoperative full blood count analysis

TKR	THR
Any of the following:	Any of the following:
Pre-op Hb of ≤ 120 g/L in female patients	Pre-op Hb of ≤ 120 g/L in female patients
Pre-op Hb of ≤ 110 g/L in male patients	Pre-op Hb of ≤ 110 g/L in male patients
Cardiac disease	Intraoperative blood loss of > 500 mL
Clinical features of anaemia postoperatively (e.g. tachycardia, hypotension)	Cardiac disease
	Clinical features of anaemia postoperatively (e.g. tachycardia, hypotension)

The criteria would have identified the only patient that required transfusion (i.e. haemoglobin < 70g/L or < 80g/L with symptoms or significant cardiac history), as well as all seven patients with post-op haemoglobin of < 90 g/L, providing a reasonable margin of error for detection of postoperative anaemia. The odds ratio of requiring transfusion in the tested group is 3.25 (95% CI 0.13-81.3, P=0.47). 

Applying Table 4 criteria to our cohort would have led to performing 25 postoperative urea and electrolyte tests (instead of 54) in the THR group and 43 postoperative urea and electrolyte tests (instead of 72) in the TKR group, saving 29 tests in each of these groups. 

**Table 4:** Proposed criteria for requesting postoperative urea and electrolyte analyses

TKR	THR
Any of the following:	Any of the following:
Pre-op Hb ≤ 120 g/L in female patients	Pre-op Hb of ≤ 120 g/L in female patients
Pre-op Hb ≤ 110 g/L in male patients	Pre-op Hb of ≤ 110 g/L in male patients
Deranged pre-op electrolytes (K < 4.0 or > 5.0 mmol/L, Na < 135or > 145 mmol/L)	Deranged pre-op electrolytes (K < 4.0 or > 5.0 mmol/L, Na < 135 or > 145 mmol/L)
Pre-op eGFR < 60 mL/min/1.73m^2^	Pre-op eGFR < 60 mL/min/.73m^2^
Cardiac disease	Cardiac or renal disease
Clinical signs or symptoms of electrolyte/renal disturbance (e.g. anuric, weakness)	Intraoperative blood loss of > 500ml
	Clinical signs or symptoms of electrolyte/renal disturbance (e.g. anuric, weakness)

Table 4 criteria would have identified all five patients that developed biochemical acute kidney injury (AKI), as well as eight of the nine patients with abnormal post-operative electrolytes. 

Overall, the criteria in both tables would have saved 59 blood tests in the cohort of 126 patients (i.e. 46.8% of patients did not require postoperative blood tests). This provides an odds ratio of 14.0 (95% confidence interval: 1.77-110, P=0.012) of an abnormal result in the patients that would have been tested, compared to those who would not have been tested. 

## Discussion

Our study identified abnormal preoperative blood tests, cardiac co-morbidities and postoperative clinic symptoms as major factors for requiring postoperative blood tests. Using our proposed criteria to determine the need for postoperative blood tests could have saved up to 47% of unnecessary tests being performed. 

There are other published studies which have evaluated the need for postoperative blood tests. Halawi et al. undertook a retrospective review of the electronic medical records of 351 patients who underwent primary unilateral total hip replacement over a two-year period [17]. Around 21% of their patients had abnormal postoperative laboratory results, of which 82.4% were exclusively due to either sodium or potassium abnormalities. The factors they found to have statistically significant associations with abnormal postoperative electrolytes were deranged serum sodium, diabetes and lack of tranexamic acid use. They found blood transfusion to be associated with higher American Society of Anaesthesiologists class and intraoperative blood loss ≥250 mL combined with either preoperative anaemia or lack of tranexamic acid use. Similarly, the factors associated with AKI in their study were higher American Society of Anaesthesiologists class and diabetes. 

The following year, Halawi et al. also published a retrospective review of 319 total knee arthroplasty procedures, similarly evaluating the factors associated with deranged postoperative blood tests [18]. They found 27.9% of the patients in their cohort had abnormal postoperative laboratory results, of which 78% were exclusively due to either sodium or potassium abnormalities. The rates of acute kidney injury (AKI) and blood transfusion were 3.8% and 1% respectively. The factors they found to be associated with electrolyte abnormalities were abnormal baseline electrolyte levels and anaemia, while the factors associated with blood transfusion were ASA score 3, preoperative anaemia, and no tranexamic acid use. The factors associated with AKI were chronic kidney disease or having at least two of the following: age >65 years, BMI >35, ASA score 3, diabetes, heart disease, and/or anaemia. There was no increased risk for 90-days hospital visits or readmissions with abnormal laboratory values. 

Howell et al. published a retrospective study which evaluated the need for full blood counts in patients who underwent total knee replacements [19]. They identified preoperative anaemia, lack of intraoperative tranexamic acid use and the female gender as major risk factors for abnormal postoperative blood tests. Surprisingly, they did not identify any comorbidity as a risk factor, while we found cardiac comorbidity as a risk factor. 

More recently, Wu et al. published the findings of their retrospective review of 395 consecutive patients who underwent primary elective total hip arthroplasty in a single centre [20]. They reported that the female gender, low body mass index, long operation time, and low preoperative haemoglobin levels were factors associated with postoperative blood transfusion. They then proposed a points-based risk-scoring system for clinical decision-making about the necessity of postoperative laboratory tests, giving specific scores to specific ranges of full blood count and electrolyte derangements. Similar to our study, the paper by Wu et al. identified abnormal preoperative blood results as significant factors for abnormal postoperative blood results, which is also in keeping with other published studies [21-23]. 

The strengths of our study include its prospective nature which allowed us closer monitoring of the patients’ hospital events such as postoperative symptoms, some of which we found to be significantly associated with the need for postoperative blood tests. In addition, as we directly monitored these patients throughout their hospital stay, we had no missing patient data. 

Unlike many of the published studies which evaluated a single procedure at a particular point in time, we evaluated both THR and TKR at the same period which allowed us direct comparison where needed, which we believe would be more reliable since both groups were exposed to the exact same conditions in the same hospital during their admission. 

Limitations

Our study has a number of limitations. First, our study evaluated relatively lower patient numbers compared to many of the published studies. Second, we only evaluated primary hip and knee procedures. However, as these are two major elective lower limb procedures, we expect our criteria can be applied to similar procedures, as well as less major and less physiologically tasking elective procedures. Third, our study was performed in a single centre and different hospital units may utilise different ranges of blood parameters.

## Conclusions

In conclusion, the ability to predict patients who are expected to have abnormal postoperative blood tests means that these patients can be identified early. This can improve patient care, especially if these identified patients are better optimised preoperatively. Avoidance of unnecessary blood analyses will also save costs and play a vital role in potential same-day discharge following primary total hip and knee arthroplasty.
